# The Effectiveness of Osteochondral Autograft Transfer in the Management of Osteochondral Lesions of the Talus: A Systematic Review and Meta-Analysis

**DOI:** 10.7759/cureus.31337

**Published:** 2022-11-10

**Authors:** Kaylem M Feeney

**Affiliations:** 1 Orthopaedics, University of Limerick School of Medicine, Limerick, IRL

**Keywords:** arthroscopy, autograft, oats, talus, olt, osteochondral

## Abstract

Osteochondral lesions of the talus (OLT) are common following ankle trauma. Operative treatment is often required, with osteochondral autografting frequently performed for large or cystic lesions, or following failed primary surgery. The aim of this systematic review was to evaluate the current evidence for osteochondral autograft transfer system (OATS) in the management of OLT. A systematic search of the PubMed, EMBASE, Scopus, and Cochrane Library databases was performed based on the Preferred Reporting Items for Systematic Reviews (PRISMA) guidelines. Study quality was assessed using the modified Coleman Methodology Score (CMS). Meta-analysis was carried out using RevMan, version 5.4 (The Cochrane Collaboration, 2020). A total of 23 studies were included. The mean modified CMS was 48.1±7.47. Fourteen studies reported preoperative and postoperative Visual Analog Scale (VAS) and American Orthopaedic Foot & Ankle Score (AOFAS). The aggregate mean preoperative and postoperative VAS score across 14 studies was 6.47±1.35 and 1.98±1.18, respectively. Meta-analysis of seven studies on 210 patients found that OATS resulted in a significant reduction in VAS score compared to baseline (Mean Difference {MD} -4.22, 95% Confidence Interval {CI} -4.54 to -3.90, *P* < 0.0001). The aggregate mean preoperative and postoperative AOFAS scores across 14 studies were 56.41±8.52 and 87.14±4.8, respectively. Based on eight studies on 224 patients, OATS resulted in a significant improvement in AOFAS score compared to baseline (MD 29.70, 95% CI 25.68 to 33.73, *P* = < 0.0001). Donor site pain occurred in 9% of cases. Current evidence from low-quality studies suggests that OATS is a safe and effective treatment option for OLT, though it is associated with a risk of donor site morbidity.

## Introduction and background

Osteochondral lesions of the talus (OLT) are frequently seen in orthopedic practice, with the majority of lesions occurring following a traumatic event, such as an ankle sprain or ankle fracture [1-6]. A consensus statement by international experts suggested that a lesion to both the cartilage and underlying subchondral bone of the talus should be referred to as an OLT [6]. There are a number of treatment options described in the literature for OLT. In the early stages, particularly in juvenile patients with intact articular cartilage, conservative treatment consisting of rest, immobilization, and non-steroidal anti-inflammatory drugs may be trialed [2,7,8]. However, conservative treatment has only been reported to be successful in approximately 54% of cases, and thus, surgical intervention is frequently required [9,10]. Surgical treatments described in the literature include arthroscopic debridement, bone marrow stimulation techniques such as microfracture, and anterograde or retrograde drilling, in addition to autologous chondrocyte implantation, autologous matrix-induced chondrogenesis or in more severe cases, osteochondral allograft transfer and osteochondral autograft transfer system (OATS) [2,10,11]. These procedures have also been augmented with biologics such as Bone Marrow Aspirate Concentrate or Platelet Rich Plasma (PRP) [2,10,11].

OATS is typically reserved for patients who either suffer large and/or cystic lesions or who have failed primary surgery [12,13]. OATS involves debridement and removal of the damaged cartilage and subchondral bone. Following the measurement of the size of the defect, the autograft is harvested most commonly from the non-weight-bearing medial or lateral femoral condyle of the ipsilateral knee. The autograft is subsequently implanted into the defect, usually through a medial (or lateral) malleolar osteotomy [3,12-14].

As far as the author is aware, just one previous systematic review investigating the effectiveness of OATS in the management of OLT has been published [11]. This review was published in 2016 and included published literature up until the point of their literature search in March 2016, with the latest study included in the review published in 2015. Unfortunately, the authors searched just two different databases, which reduces the sensitivity of their search for observational studies from approximately 90% to approximately 65% [15]. Therefore, it is possible that a number of studies may have been missed during their search. In addition, they did not perform a meta-analysis of their results. Finally, since the publication of that systematic review, a number of studies have been performed and published [14,16-25]. Therefore, the aim of this systematic review and meta-analysis was to evaluate the current evidence for the use of OATS in the management of OLT.

## Review

Materials and methods

Search Strategy

This systematic review was carried out in accordance with the updated Preferred Reporting Items for Systematic Reviews (PRISMA) guidelines [26]. A comprehensive search of the literature was performed using the databases PubMed, EMBASE, Scopus, and the Cochrane Library. As recommended in the literature, four databases were chosen to improve the sensitivity of the search [15,27]. The search terms used were: '(OATS OR osteochondral autograft transfer system OR osteochondral autografting OR mosaicplasty OR autologous osteochondral transplant)’ AND ‘(ankle OR talus OR “osteochondral lesion of the talus’ OR OLT). These search terms were developed in line with international expert consensus on terminology for OLT [6]. Databases were searched for articles published between 1st June 2012 and 31st May 2022 to ensure the most current research was included. Reference lists of included studies were also manually searched.

Inclusion and exclusion criteria are specified in Table 1. Search results of each database were exported to the systematic review manager Rayyan (http://rayyan.qcri.org) for the title and abstract screening [28]. Titles and abstracts were screened and following screening and removal of duplicates, the remaining articles were retrieved for full-text review and compared to the inclusion and exclusion criteria. Articles that did not meet inclusion criteria were excluded.

**Table 1:** Inclusion and Exclusion Criteria OLT: Osteochondral lesions of the talus; OATS: Osteochondral autograft transfer system

Inclusion Criteria
Evidence level I-IV studies evaluating the clinical outcomes of OATS procedure for OLT.
Minimum sample size of 10 patients.
Minimum follow-up period of 6 months.
Published in a peer-reviewed journal between 1^st^ June 2012 and 31^st^ May 2022.
Published in the English language.
Exclusion Criteria
Systematic review or level V study.
Published outside of the timeframe specified above.
Published in a language other than English.

Data Extraction

The following details of interest were extracted from each study: name of authors, year of publication, study type, level of evidence, number of patients, number of lesions, percentage of patients attending final follow-up, and duration of follow-up. The level of evidence was assessed according to the five levels of evidence [29]. Patient demographics including gender, age, duration of symptoms, history of trauma, lesion location, lesion size, and history of prior ankle surgery were also extracted. Finally, details regarding outcome measures, surgical approach, complications/failures, donor site location, and donor site morbidity were recorded.

Methodological Quality

The methodological quality of included studies was formally assessed using the modified Coleman Methodology Score (CMS) [30]. The modified version of the original CMS [31] was created to specifically assess the quality of studies on cartilage repair. The score uses 10 specific criteria to formally assess study quality and has been used in a number of similar studies [11,30,32,33]. Each study is given a score for each section, with a final score ranging between 0 (worst quality) and 100 (highest quality).

Statistical Analysis

A meta-analysis was performed on non-comparative studies to evaluate the effectiveness of OATS. Meta-analysis was not performed on comparative studies due to the small number of studies (n = 4) and heterogeneity between study methodology and outcomes. Meta-analysis was performed using Review Manager (RevMan) (a computer program), version 5.4 (The Cochrane Collaboration, 2020). Only clinical outcome measures that were utilized in five or more studies, and reported preoperative and postoperative mean and standard deviation (SD) of scores were included. As these are continuous variables, results were expressed as mean difference (MD) and confidence interval (CI), which was set at 95%. Heterogeneity between studies was quantified using the I^2^ statistic [34]. A *P *value of <0.05 was considered statistically significant.

Results

Search Results

**Figure 1:**
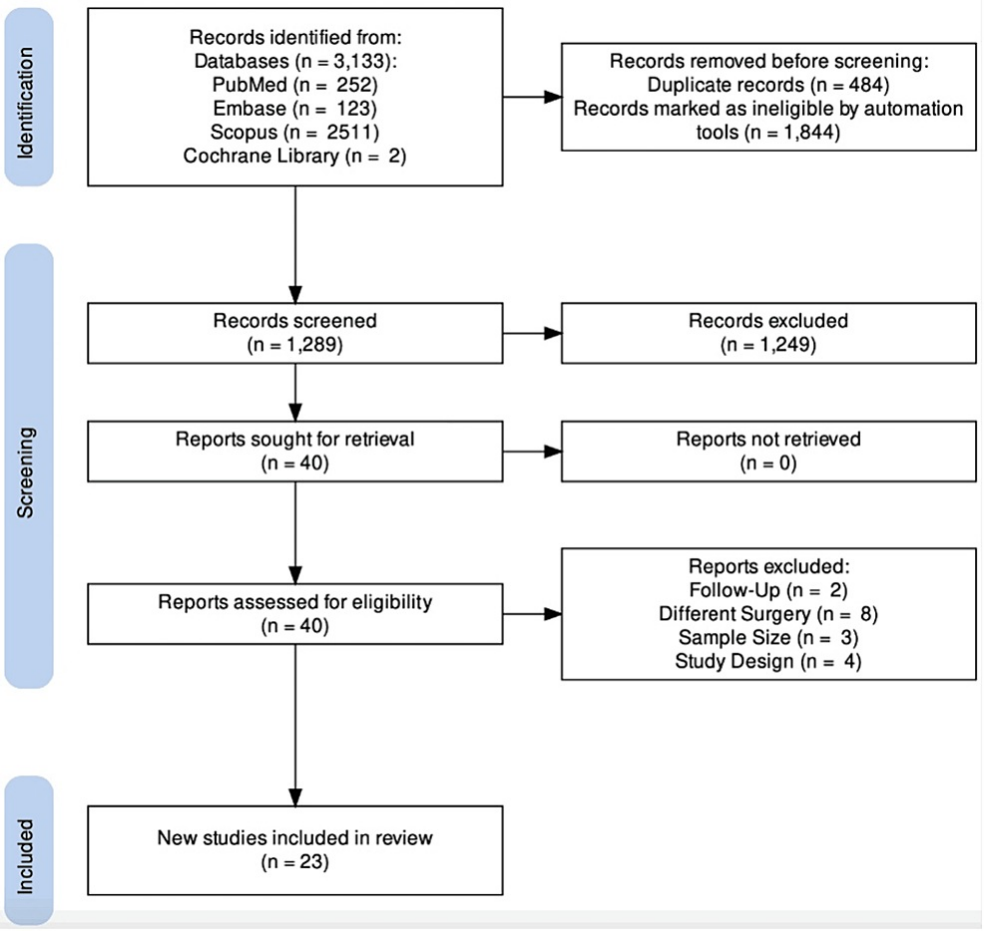
PRISMA Flow Chart PRISMA: Preferred Reporting Items for Systematic Reviews

Following a search of the literature, a total of 3,133 articles were identified (Figure 1). Following the removal of duplicates and the application of filters, 1,289 articles were screened for eligibility. Of these 1,289 articles, 1,249 were excluded following the application of inclusion and exclusion criteria. A total of 40 articles were retrieved for full-text review. A further 17 articles were excluded following a full-text review. A search of the reference lists of full-text articles yielded no additional articles. A total of 23 articles including 797 patients were included in this study (Figure 1).

Demographic Data and Patient History

Demographic data are summarised in Table 2. Across the 23 studies, a total of 797 patients were included in this review. Of the 797 patients, 527 (66.1%) were male, while 270 (33.9%) were female. The mean age was 36.2±7.06 years (range 25.4 - 55.4). The mean duration of symptoms prior to surgery, reported in 14 studies, was 21.66±13.94 months. The mean area of OLT, as reported in 13 studies, was 135.5±45.85mm^2^ (range 85 - 249mm^2^). Out of 12 studies on 470 patients, 344 (73.2%) reported a history of trauma. One study (4.3%) reported that no patients included had prior ankle surgery before undergoing OATS, while nine studies (39.1%) did not provide this data. Across the remaining 13 studies, 243 of 476 (51%) patients had undergone ankle surgery prior to OATS.

**Table 2:** Patient Demographics and Patient History M = Male; F = Female; NR = not reported

Author	Gender	Mean Age (years)	Mean Duration of Symptoms (months)	History of Trauma [n (%)]	Mean Area of Lesion (mm^2^)	Prior Ankle Surgery [n (%)]
Guney et al. [3]	M = 32; F = 22	40.1	8.9	43 (80)	NR	NR
Haleem et al. [12]	M = 24; F = 18	43.7	31.8	29 (69)	118	NR
Shimozono et al. [13]	M = 19; F = 22	38.4	28.1	NR	116.2	16 (39)
Adanas and Ozkan [15]	M = 15; F = 8	32.3	13	17 (73.9)	Diameter Only	NR
Bai et al. [16]	M = 14; F = 5	33	NR	NR	NR	NR
Basal and Aslan [17]	M = 8; F = 3	31.7	6	5 (45)	Diameter Only	No
de L’Escalopier et al. (2021) [18]	M = 37; F = 19	34	21	42 (75)	Diameter Only	11 (20)
Kim and Haskell [20]	M = 11; F = 21	48.6	NR	NR	86.2	3 (9)
Li et al. [21]	M = 6; F = 7	55.4	NR	6 (46)	135.9	NR
Toker et al. [22]	M = 11; F = 9	33.5	21.9	NR	Diameter Only	4 (20)
Park et al. [24]	M = 31; F = 15	34.2	NR	NR	194.9	28 (61)
Ahmad and Jones [34]	M = 21; F = 15	41.3	NR	NR	160	27 (75)
Yoon et al. [35]	M = 33; F = 11	37.1	59.9	26 (59)	152.9	44 (100)
Petersen et al. [36]	M = 12; F = 8	25.4	NR	NR	Diameter Only	20 (100)
de L’Escalopier et al. (2015) [37]	M = 29; F = 8	33	29	31 (84)	85	8 (22)
Flynn et al. [38]	M = 55; F = 30	36.7	29.6	52 (59.8)	104.2	NR
Nguyen et al. [39]	M = 38; F = 0	26	9.7	NR	249	13 (34)
Georgiannos et al. [40]	M = 37; F = 9	36.2	12	46 (100)	NR	46 (100)
Wan et al. [41]	M = 16; F = 8	39.1	19.6	NR	NR	17 (70.8)
Zhang et al. [42]	M = 14; F = 9	31.4	12.7	16 (70)	113.2	NR
Sabaghzadeh et al. [43]	M = 11; F = 8	43	NR	NR	NR	NR
Fraser et al. [44]	M = 24; F = 12	31	NR	31 (86)	133	6 (17)
Emre et al. [45]	M = 29; F = 3	27.5	NR	NR	113	NR

Study Design

Of the 23 studies included, 17 (73.9%) were level IV studies, while four (17.4%) were level III and two (8.7%) were level II studies (Table 3). The mean duration of follow-up across all studies was 47.7±32.68 months (range 12 - 143.5 months). The mean effective percentage of follow-up was 96.46% (range 76 - 100%). All 23 studies (100%) adequately described their surgical technique. A total of 22 out of 23 studies adequately described their postoperative protocol. The only study that did not describe their postoperative protocol was Toker et al. [23] Of the 23 studies included, 19 (82.6%) were non-comparative, while four (17.4%) were comparative studies [3,14,35,36].

**Table 3:** Characteristics of Included Studies *Comparative Study

Author	Study Type	Level of Evidence	Number of Patients	Number of Lesions	Effective Follow-Up (%)	Mean Duration of Follow-Up (Months)
Guney et al. [3]	Non-Randomised Control Trial	II	54	54	100	42
Haleem et al. [12]	Prospective Cohort	III	42	42	100	85
Shimozono et al. [13]	Case Control	III	41	41	100	26.3
Adanas and Ozkan [15]	Retrospective Series	IV	23	23	100	12
Bai et al. [16]	Retrospective Series	IV	19	19	76	32.5
Basal and Aslan [17]	Prospective Series	IV	11	11	100	13
de L’Escalopier et al. (2021) [18]	Retrospective Series	IV	56	56	87.5	102
Kim and Haskell [20]	Prospective Series	IV	32	33	100	19.5
Li et al. [21]	Retrospective Series	IV	13	13	85	21.2
Toker et al. [22]	Retrospective Series	IV	20	21	83	143.5
Park et al. [24]	Prospective Cohort	III	46	46	100	72
Ahmad and Jones [34]	Comparative Series	II	36	36	90	35.2
Yoon et al. [35]	Prospective Cohort	III	44	44	100	45
Petersen et al. [36]	Prospective Series	IV	20	20	100	25.8
de L’Escalopier et al. (2015) [37]	Retrospective Series	IV	37	37	100	76
Flynn et al. [38]	Retrospective Series	IV	85	87	100	47.2
Nguyen et al. [39]	Retrospective Series	IV	38	38	97	44.7
Georgiannos et al. [40]	Retrospective Series	IV	46	48	100	66
Wan et al. [41]	Prospective Series	IV	24	24	100	50.9
Zhang et al. [42]	Retrospective Series	IV	23	23	100	37.1
Sabaghzadeh et al. [43]	Retrospective Series	IV	19	19	100	12
Fraser et al. [44]	Retrospective Series	IV	36	36	100	71
Emre et al. [45]	Prospective Series	IV	32	32	100	16.8

Quality Assessment

The mean modified CMS [30] across all 23 studies was 48.1±7.47 (range 32 - 61), suggesting that the studies included were of poor to average quality. The study with the highest methodological quality, with a modified CMS of 61/100, was published by Ahmad and Jones in 2015 [35]. The mean modified CMS across the four comparative studies was 54.25±2.99 (range 54 - 61), which was higher than the overall mean. The areas with the most significant methodological deficiencies across studies were sample size, study design, outcome assessment, and subject selection.

Clinical Outcomes

The clinical outcome measures used in each study are highlighted in Table 4. The most frequently used outcome measure was the American Orthopaedic Foot & Ankle Score (AOFAS) score, used in 69.5% (n = 16) of studies (Table 4). The VAS score was reported in 65.2% (n = 15) of studies (Table 4). A total of 26% (n = 6) of studies reported Foot and Ankle Outcome (FAOS) scores, while the Short Form Survey (SF-12) score was reported in 8.7% (n = 2) of studies. Both the Foot and Ankle Ability Measure (FAAM) score and the Ogilvie-Harris score were reported in 4.3% (n = 1) of studies (Table 4).

**Table 4:** Clinical Outcomes VAS = Visual Analog Scale; AOFAS – American Orthopaedic Foot & Ankle Society Score; FAOS = Foot and Ankle Outcome Score; FAAM = Foot and Ankle Ability Measure; SF-12 = 12 Item Short Form Survey; NR = not reported; *Statistically significant (P = <0.05); **Ogilvie Harris Score rated good-excellent in 29/37 (78%) of cases.

Author	Outcome Measure 1	Preoperative Value [Mean (Range)]	Postoperative Value [Mean (Range)]	Outcome Measure 2	Preoperative Value [Mean (Range)]	Postoperative Value [Mean (Range)]
Guney et al. [3]	VAS	7.8 (NR)	2.1 (NR)*	AOFAS	43.8 (NR)	77.3 (NR)*
Haleem et al. [12]	FAOS	51.55 (NR)	87.06 (NR)*	SF-12	49.46 (NR)	86.14 (NR)*
Shimozono et al. 13]	FAOS	46.1 (43 – 49.2)	81.9 (78.6 – 85.2)*	SF-12	39.8 (35.9 – 43.7)	74.7 (71 – 78.4)*
Adanas and Ozkan [15]	VAS	7.39 (NR)	2.04 (NR)*	AOFAS	55.65	88.91*
Bai et al. [16]	VAS	4.68 (NR)	0.47 (NR)*	AOFAS	72.8 (NR)	93.7 (NR)*
Basal and Aslan [17]	AOFAS	49.3 (NR)	86.1 (NR)*			
de L’Escalopier et al. (2021)[18]	AOFAS	NR	80.6 (NR)	FAOS	NR	77.8 (NR)
Kim and Haskell [20]	VAS	4.7 (NR)	1.4 (NR)*	AOFAS	65.4 (NR)	86.9 (NR)*
Li et al. [21]	VAS	6.7 (NR)	1.9 (NR)*	AOFAS	53 (NR)	90 (NR)*
Toker et al. [22]	VAS	6.3 (5 – 7)	2 (0 – 4)*	AOFAS	60.4 (48 – 70)	86.2 (60 – 94)*
Park et al. [24]	VAS	6.2 (NR)	1.9 (NR)*	FAOS	NR	NR*
Ahmad and Jones [34]	VAS	7.9 (NR)	2.2 (NR)*	FAAM	54.5 (31 – 88.1)	85.5 (56 – 97.6)*
Yoon et al. [35]	VAS	6.14 (NR)	1.91 (NR)*	AOFAS	50.4 (NR)	85.27 (NR)*
Petersen et al. [36]	VAS	NR	NR*			
de L’Escalopier et al. (2015) [37]	AOFAS	NR	83 (9-100)	Ogilvie-Harris	NR	Good/Excellent (78%)**
Flynn et al. [38]	FAOS	50.3 (2.8 – 86.1)	81 (69.4 -100)*			
Nguyen et al. [39]	VAS	4.53 (NR)	0.63 (NR)*	FAOS	NR	NR*
Georgiannos et al. [40]	VAS	9.1 (NR)	5.2 (NR)*	AOFAS	55 (NR)	91 (NR)*
Wan et al. [41]	VAS	6.1 (NR)	2 (NR)*	AOFAS	61.3 (NR)	84.9 (NR)*
Zhang et al. [42]	VAS	5.6 (4 – 7)	0.7 (0 – 3)*	AOFAS	56 (40 – 78)	93.8 (83 – 100)*
Sabaghzadeh et al. [43]	VAS	7.4 (NR)	3.2 (NR)*	AOFAS	42.1 (NR)	78.6 (NR)*
Fraser et al. [44]	AOFAS	65.5 (NR)	89.4 (NR)*			
Emre et al. [45]	AOFAS	59.12 (NR)	87.94 (NR)*			

**Figure 2:**
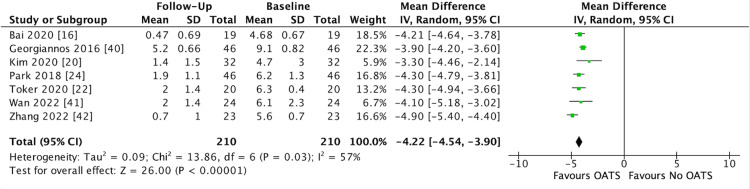
Forest Plot Depicting Meta-Analysis of VAS Score SD = Standard Deviation; CI = Confidence Interval; VAS = Visual Analog Scale Source: References [16,20,22,24,40-42]

Of the 15 studies reporting VAS scores, 14 reported preoperative and postoperative scores, one did not report the specific VAS score [37]. Of the 15 studies included reporting VAS scores, seven reported mean (SD) and thus were included in the meta-analysis (Figure 2). The aggregate mean preoperative and postoperative VAS scores across the 14 studies was 6.47±1.35 (range 4.53 - 9.1) and 1.98±1.18 (range 0.47 - 5.2), respectively (Table 4). All 14 studies reported that the mean reduction in VAS score from baseline to follow-up was statistically significant (P < 0.05). The one study which did not report specific VAS scores did state that the mean VAS reduction was statistically significant [37]. The meta-analysis in this study found that based on seven studies on 210 patients at final follow-up, OATS resulted in a significant reduction in VAS score compared to baseline (MD -4.22, 95% CI -4.54 to -3.90, I^2^ = 57%, *P *< 0.0001) (Figure 2).

**Figure 3:**
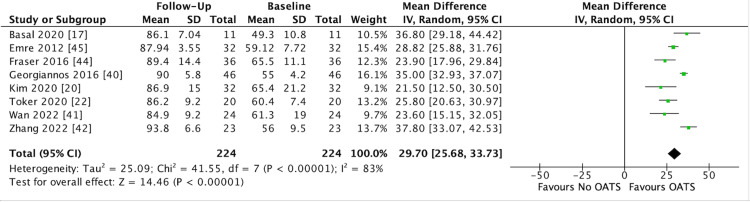
Forest Plot Depicting Meta-Analysis of AOFAS Score SD = Standard Deviation; CI = Confidence Interval; AOFAS = American Orthopaedic Foot & Ankle Score Source: References [17,20,22,40-42,44,45]

Of the 16 studies reporting AOFAS scores, 14 reported preoperative and postoperative scores, while two reported postoperative scores only [19,38]. Of these 16 studies, eight reported mean (SD) scores and were therefore included in the meta-analysis (Figure 3). The aggregate mean preoperative and postoperative AOFAS score across the 14 studies was 56.41±8.52 (range 42.1 - 72.8) and 87.14±4.8 (range 77.3 - 93.8), respectively (Table 4). All 14 studies reported that the mean improvement in AOFAS score from baseline to follow-up was statistically significant (*P* < 0.05). The meta-analysis in this study found that based on eight studies on 224 patients at final follow-up, OATS resulted in a significant improvement in AOFAS score compared to baseline (MD 29.70, 95% CI 25.68 to 33.73, I^2^ = 83%, *P* = < 0.0001) (Figure 3).

Of the six studies reporting FAOS scores, three reported preoperative and postoperative scores (Table 4). These three studies both observed a significant improvement in FAOS score compared to baseline (*P* <0.05) [12,14,39]. Two of the five studies did not report any FAOS values, while one reported the postoperative FAOS score only [19,25,40]. Both studies that reported SF-12 scores reported preoperative and postoperative scores, with both studies observing a significant improvement in from baseline to final follow-up [12,14].

Of the four comparative studies included in this review, two compared the effectiveness of OATS versus allograft in the management of OLT [14,35]. Ahmad and Jones [35] observed no significant difference in either mean VAS (7.9 to 2.2 versus 7.8 to 2.7; *P* > 0.05) or FAAM (54.4 to 85.5 versus 55.2 to 80.7, *P* > 0.05) scores between the OATS and allograft group from baseline to follow-up, respectively, suggesting that OATS and allograft have comparable results. In contrast, Shimozono et al. [14] reported that in their study, OATS resulted in a significantly higher postoperative mean FAOS (81.9 versus 70.1, *P* = 0.006) and SF-12 scores (74.7 versus 66.1, *P* = 0.021) when compared to allograft, suggesting that OATS is superior to the allograft.

One study compared the outcomes of OATS versus repeated microfracture in the management of OLT, in patients who had failed primary treatment with microfracture [36]. The authors reported that OATS resulted in significantly superior VAS (1.91 versus 5.27, *P* = <0.001) and AOFAS scores (85.27 versus 69.64, *P* = <0.001) when compared to repeated microfracture at follow-up.

The final comparative study included compared the effectiveness of primary OATS versus primary arthroscopic microfracture with or without PRP in the management of OLT [3]. The authors reported no significant difference in mean AOFAS score in the OATS, microfracture, or microfracture with PRP groups (77.3 versus 73.1 versus 75.4, respectively, *P* = >0.05) at the final follow-up. However, the authors did report a significant difference in the mean change in VAS score in the OATS group compared to the microfracture and microfracture with PRP groups (-5.8 versus -3.2 versus -4.3, respectively, *P* = 0.023).

Surgical Approach and Complications

A total of 13 (56.5%) studies reported a total of 53 complications or required further surgery (Tables 5, 6). The most frequent complication was the development of soft tissue impingement requiring arthroscopic debridement (n = 17; 32%). This was followed by cyst formation at the operative site (n = 13; 24.5%) and screw removal due to pain at the medial malleolar osteotomy site (n = 7; 13.2%). Two studies removed all medial malleolar hardware but did not state whether this was routine or was indicated for pain [25,37]. A total of 11 studies (47.8%) performed a medial malleolar osteotomy on all patients, while eight studies (34.8%) performed either a medial or lateral malleolar osteotomy depending on lesion location. One study (4.3%) performed an anterior malleolar osteotomy, while two studies (8.7%) performed either an anterior or medial malleolar osteotomy depending on OLT location (Table 5).

**Table 5:** Clinical Variables in Included Studies MMO = Medial Malleolar Osteotomy; LMO = Lateral Malleolar Osteotomy; AMO = Anterior Malleolar Osteotomy; NR = Not reported; * = posterior femoral condyle.

Author	Surgical Approach	Complications/ Failures	Donor Site	Donor Site Pain [n (%)]
Guney et al. [3]	MMO	No	Ipsilateral knee	2 (15)
Haleem et al. [12]	MMO	No	Ipsilateral knee	2 (4.8)
Shimozono et al. [13]	MMO	1	Ipsilateral knee	1 (4)
Adanas and Ozkan [15]	MMO	No	Ipsilateral knee	0
Bai et al. [16]	AMO/MMO	No	Ipsilateral knee	0
Basal and Aslan [17]	AMO	No	Ipsilateral knee	3 (27)
de L’Escalopier et al. (2021) [18]	MMO/LMO	No	Ipsilateral knee	11 (20)
Kim and Haskell [20]	Arthrotomy	2	Ipsilateral distal tibia	0
Li et al. [21]	MMO	2	Ipsilateral knee	0
Toker et al. [22]	MMO	2	Ipsilateral knee	3 (15)
Park et al. [24]	MMO	11	Ipsilateral knee	NR
Ahmad and Jones [34]	MMO/LMO	2	Ipsilateral knee	6 (30)
Yoon et al. [35]	MMO	No	Ipsilateral knee	4 (18)
Petersen et al. [36]	MMO/LMO	1	Ipsilateral knee*	0
de L’Escalopier et al. (2015) [37]	MMO/LMO	No	Ipsilateral knee	6 (16)
Flynn et al. [38]	MMO/LMO	7	Ipsilateral knee	2 (2.4)
Nguyen et al. [39]	MMO/LMO	3	Ipsilateral knee	2 (5.3)
Georgiannos et al. [40]	AMO/MMO	No	Ipsilateral talus	0
Wan et al. [41]	MMO	15	Ipsilateral talus	0
Zhang et al. [42]	MMO	1	Ipsilateral talus	0
Sabaghzadeh et al. [43]	MMO/LMO	3	Ipsilateral knee	NR
Fraser et al. [44]	MMO/LMO	3	Ipsilateral knee	4 (11)
Emre et al. [45]	MMO	No	Ipsilateral knee	2 (6)

**Table 6:** Description and Frequency of Complications and Requirement for Subsequent Surgery

Complication	Frequency [n (%)]
Arthroscopic Debridement for Soft Tissue Impingement	17 (32)
Bone cyst	13 (24.5)
Medial malleolar hardware removal	7 (13.2)
Ankle Arthrodesis	3 (5.6)
Delayed union	3 (5.6)
Repeat OATS	2 (3.8)
Infection	2 (3.8)
Calcaneus pain	2 (3.8)
Broken graft	1 (1.9)
Supramalleolar osteotomy	1 (1.9)
Deep vein thrombosis	1 (1.9)
Steroid injection (knee)	1 (1.9)

A total of 18 (78.3%) studies harvested the autograft from the non-weight-bearing part of either the medial or lateral femoral condyle of the ipsilateral knee, while three studies (13%) obtained the autograft from the ipsilateral talus. One study (4.3%) harvested the graft from the posterior femoral condyle of the ipsilateral knee, while one study (4.3%) harvested the autograft from the ipsilateral distal tibia (Table 5).

The incidence of donor site pain is summarised in Table 5. No donor site pain was reported from any patient in the three studies that harvested the autograft from the ipsilateral talus, nor in the one study that harvested the graft from the ipsilateral distal tibia [41-44]. Among the 19 studies that harvested the autograft from the ipsilateral knee, two did not report on donor site pain [25,45], while four reported zero cases of donor site pain, including one which harvested the autograft from the posterior femoral condyle [16,17,22,37]. Across the remaining 13 studies that harvested the autograft from the ipsilateral knee, a total of 48 out of 532 (9%) patients reported donor site pain at the final follow-up.

Discussion

OLT is a common injury of the ankle. Conservative management frequently fails and thus, operative management is often required [9,10]. The range of surgical options for managing OLT is well described in the literature [2]. This systematic review is a synthesis of 23 studies evaluating the effectiveness of OATS in the management of OLT. Of the 23 studies, 17 were level IV, four were level III and two were level II studies (Table 3) published across a ten-year period.

Demographics

The majority of patients in this study were male (527/797; 66.1%) and aged between 25 and 55 years (Table 2). Considering the mean duration of symptoms prior to surgery was 21.66 months, it is clear that OLT can have a significant social and economic impact on patients, with many patients potentially missing out on work due to pain. These findings are consistent with previous studies [2,11,46].

Trauma has been well described as a primary aetiologic factor in the development of OLT [2]. In this study, across 470 patients in studies reporting a traumatic history, 73.2% (344/470) of patients had a history of trauma. This is consistent with other studies [2,11,47]. This highlights the fact that clinicians should have a high index of suspicion for OLT if a patient has persistent pain following any ankle trauma (e.g. ankle sprain). Across 13 studies, 243 of 476 (51%) had prior ankle surgery before undergoing OATS, suggesting that OATS is still viewed by many clinicians as a procedure used following failed primary surgery, with the exception of large or cystic lesions. This is consistent with previously published studies and recommendations of a systematic review published in 2010 [2,10,11].

The mean area of OLT in this study was 135.5mm^2^ (range 85 - 249mm^2^), which is interesting considering that OATS is most commonly used for larger lesions >150mm^2^, though this has been identified in prior studies and may reflect the fact that OATS is often used as a secondary procedure for smaller lesions following failed primary surgery [11].

The most important findings from this review are that at a mean follow-up of 47.7±32.68 months across all studies, excellent clinical outcomes were demonstrated (Table 4). The most frequently reported clinical outcome measures were the AOFAS score (n = 16) and the VAS score (n = 15). The meta-analysis, which pooled data from seven studies for VAS and eight studies for AOFAS, demonstrated that significant improvement in symptoms is achieved following OATS for OLT [17,18,23,41,42,43,44,48,49]. The meta-analysis demonstrated that a mean VAS reduction of 4.22 (95% CI -4.54 to -3.90) can be expected following OATS for OLT (Figure 2). In addition, an improvement in AOFAS score by a mean of 29.7 (95% CI 25.68 to 33.73) can be expected following OATS (Figure 3). As the first meta-analysis to be performed on studies evaluating OATS for OLT, these findings are important for clinicians. All 21 studies that measured pain and functional outcomes preoperatively and postoperatively demonstrated a statistically significant improvement in outcome measures for OATS for OLT (Table 4). The findings highlight the consistent benefits of OATS for OLT, which were observed across all studies.

Comparative Studies

Two comparative studies have suggested that, in terms of clinical outcomes, OATS is at least comparable to allograft in the management of OLT [14,35]. However, further higher-quality studies are required to confirm this. Two other comparative studies have compared OATS to repeat microfracture, and to microfracture with and without PRP [3,36]. Both of these studies provide evidence to suggest that OATS is at least comparable to microfracture as a primary surgery, and is superior to repeat microfracture as a secondary procedure. This also confirms the current general consensus that OATS is an excellent option as a secondary procedure following failed microfracture [39].

Across the 23 studies, with the exception of large cystic lesions, OATS was performed most frequently as a secondary procedure following failed primary surgery (e.g. microfracture). However, excellent outcomes were reported at a mean follow-up of 42 months in the one study that performed OATS as a primary procedure [3]. This suggests that OATS is effective as both a primary and secondary procedure.

Surgical Technique and Donor Site Morbidity

The surgical technique was well described in all 23 studies, with the most common surgical approach requiring a medial malleolar osteotomy to obtain access to the OLT (Table 5). This finding is consistent with previous studies and highlights the fact that most OLT occur medially [2,11,44]. Interestingly, one study performed OATS through an arthrotomy without an osteotomy and reported favorable results, suggesting that many lesions, particularly anterior lesions, may be accessed without the necessity of an osteotomy [44]. Donor site morbidity was observed in a total of 48 out of 532 (9%) patients at the final follow-up (Table 5). This is consistent with the findings of other studies in this area, including a meta-analysis that reported donor site morbidity in 6.7 - 10.8% of cases following OATS for OLT [49]. This is an important aspect of OATS that should be discussed with patients prior to surgery. Interestingly, the three studies, on 93 patients who harvested the autograft from the ipsilateral talus, reported no patients had donor site pain at follow-up, suggesting that autograft harvested from the talus may reduce the incidence of donor site pain compared to the ipsilateral knee, though larger comparative studies are required to confirm this [41-43].

Strengths and Limitations

One limitation of this study is the exclusion of studies in a language other than English potentially introduces language bias. Strengths of this study include the systematic search of four major databases, in addition to the use of a quality assessment tool to formally assess the methodological quality of included studies.

## Conclusions

The results of this systematic review and meta-analysis suggest that OATS is a safe and effective procedure for managing OLT. It results in excellent clinical outcomes even at long-term follow-up. This review suggests that OATS consistently reduces pain and improves function in those with OLT. While most studies included in this review performed OATS as a secondary procedure, the best available evidence suggests that OATS is effective as both a primary procedure and as a secondary procedure following failed primary surgery (e.g. microfracture). Patients should be counseled prior to surgery to ensure they understand that based on our systematic review, there is approximately a 9% risk of donor site pain when the autograft is harvested from the ipsilateral knee. Further high-quality comparative studies are required to confirm these findings and compare OATS to alternative procedures such as allograft. In addition, further comparative studies are required to determine the optimal site for autograft harvest given the risk of donor site pain when autograft is harvested from the ipsilateral knee.
